# Spatial representations of place cells in darkness are supported by path integration and border information

**DOI:** 10.3389/fnbeh.2014.00222

**Published:** 2014-06-24

**Authors:** Sijie Zhang, Fabian Schönfeld, Laurenz Wiskott, Denise Manahan-Vaughan

**Affiliations:** ^1^Department of Neurophysiology, Medical Faculty, Ruhr University BochumBochum, Germany; ^2^International Graduate School for Neuroscience, Ruhr University BochumBochum, Germany; ^3^Institute for Neural Computation, Ruhr University BochumBochum, Germany

**Keywords:** sensory, hippocampus, CA1, place cells

## Abstract

Effective spatial navigation is enabled by reliable reference cues that derive from sensory information from the external environment, as well as from internal sources such as the vestibular system. The integration of information from these sources enables dead reckoning in the form of path integration. Navigation in the dark is associated with the accumulation of errors in terms of perception of allocentric position and this may relate to error accumulation in path integration. We assessed this by recording from place cells in the dark under circumstances where spatial sensory cues were suppressed. Spatial information content, spatial coherence, place field size, and peak and infield firing rates decreased whereas sparsity increased following exploration in the dark compared to the light. Nonetheless it was observed that place field stability in darkness was sustained by border information in a subset of place cells. To examine the impact of encountering the environment’s border on navigation, we analyzed the trajectory and spiking data gathered during navigation in the dark. Our data suggest that although error accumulation in path integration drives place field drift in darkness, under circumstances where border contact is possible, this information is integrated to enable retention of spatial representations.

## Introduction

In the hippocampal formation, four neuronal populations enable the encoding of spatial representations. Place cells are found in the CA1 region of the hippocampus (O’Keefe and Dostrovsky, 1971) and fire when an animal finds itself in a specific location of an environment. Head direction cells (located e.g., in the postsubiculum) exhibit a high firing rate when an animal’s head points to a specific direction (Taube et al., 1990) regardless of the location of the animal in an environment. Border vector cells (Barry et al., 2006; Lever et al., 2009) and border cells (Solstad et al., 2008) (found e.g., in the subiculum and medial entorhinal cortex) encode the animal’s location relative to the borders or boundaries of the environment. Finally, grid cells occurring in the medial entorhinal cortex exhibit firing fields that define a triangular array representing the entire perceived environment (Hafting et al., 2005). All of these spatially selective firing patterns derive from external information in the form of stable sensory cues (Muller and Kubie, 1987; Save et al., 2000; Zhang and Manahan-Vaughan, 2013) and from internal information such as self-motion generated path integration (McNaughton et al., 1989; Quirk et al., 1990).

Path integration is a process that was originally postulated to keep track of positions relative to a home base, by updating a stored vector pointing to this location. This term has come to be used to refer not only to a process used in homing behavior when foraging animals return “home” immediately after finding a piece of food, but also to refer to an automatic and constant function when animals move in space. This latter process serves to maintain a map-like spatial representation using initial reference and self-motion cues (McNaughton et al., 1996, 2006; Etienne and Jeffery, 2004). It allows the animal to estimate its current location by integrating changes in distance and direction from the original position: a form of dead reckoning. Internal path integration and external sensory perception are two complementary mechanisms in spatial navigation. In darkness in the absence of visual input, the maintenance of accurate path integration depends on rapid updating by means of external sensory information from the ambient environment. This external sensory input can take the form of an odor cue on a nearby wall (Goodridge et al., 1998) that stabilizes head direction cell firing, can derive from self-generated odor cues left behind during previous exploration (Save et al., 2000), or can be spatially arranged odor constellations (Zhang and Manahan-Vaughan, 2013) that stabilize place cell firing. In addition to odor cues, somatosensory cues that provide a physical point of reference can also be used as an anchor, such as an object in the environment (Rochefort et al., 2011), that provides a physical point of reference.

Without anchors of this kind, firing patterns of spatial representations such as head direction cells and place cells rapidly degrade (Mizumori and Williams, 1993; Goodridge et al., 1998). For example, place fields have been reported to become unreliable over a period of 48 min in darkness, but if the recording environment is not cleaned to remove odor reference points, over half of the place fields remain relatively stable (Save et al., 2000). Nevertheless, a moderate dispersion of place cell firing was observed in the dark whether reliable odor cues were present or not. It is widely recognized that such degradation of spatial firing pattern is due to a progressive accumulation of error in the path integration system under circumstances where external sensory cues are not salient enough to be used as spatial anchors. To date, few studies have focused on quantitative analysis of the error accumulation in path integration in the dark.

Border information is also important in spatial navigation (Barry et al., 2006; Solstad et al., 2008; Lever et al., 2009). Although external sensory inputs are minimized in darkness, animals navigating in an enclosure are still able to sense the border. We postulate that border information, in the form, for example, of visual or olfactory information, is processed in helping to reduce error accumulation in path integration. In this study, we recorded from place cells in navigating animals under three conditions, lights on—lights off—lights on again, in an open field circular arena that contained no physical orientation cues and where sensory cues were suppressed. Place cells were recorded through all trials. We observed that place fields drift during darkness but that collisions with the border walls help to rectify error accumulation.

## Materials and methods

### Subjects

The present study was carried out in accordance with the European Communities Council Directive of September 22nd, 2010 (2010/63/EU) for care of laboratory animals. All experiments were performed according to the guidelines of the German Animal Protection Law and were approved by the North Rhine-Westphalia State Authority (Bezirksamt, Arnsberg). All efforts were made to reduce the number of animals used.

Male Wistar rats (8–9 weeks old) were housed individually and maintained on a 12-h light/12-h dark cycle. The animals were given sufficient food to maintain 90% of their free-feeding weight and *ad libitum* access to water. They were handled individually for 10 min per day, 1 week before surgery.

### Electrodes and microdrives

One lightweight microdrive (Axona Ltd, St. Albans, UK) was chronically implanted in each rat (8–9 weeks at the time of surgery). Each microdrive held four tetrodes, made of four twisted bundles of Formvar-coated electrodes (25 µm) platinum-iridium wires (A-M systems, USA). Each tetrode was strengthened with cyanoacrylate glue and inserted into a cannula, which was attached to the microdrive. One full rotation of the mechanical drive produces a vertical movement of 200 µm without rotating the cannula or the electrodes.

### Surgery

Each rat was chronically implanted with a microdrive as follows: Animals were anesthetized with an initial dose of sodium pentobarbital (52 mg/kg, i.p.) and placed in a stereotactic unit. Body temperature was monitored throughout the operation and the anesthetic dose was adjusted to maintain surgical anesthesia. A hole was drilled (1.2 mm diameter) over the right hippocampus. The tetrodes were placed in the cortex just above the CA1 hippocampal subfield (bregma −3.8 mm AP, 3.0 mm ML and 1.5 mm DV). To protect the exposed part of the tetrodes between the skull surface and the bottom of the cannula, a sleeve made of 19-gauge tubing was pulled down over the exposed tetrodes to a depth just below the skull surface, the top of which overlapped the cannula. Three holes were drilled in the frontal, parietal and occipital bone respectively into which small jewelers’ screws were inserted. The microdrive was then anchored to the jewelers’ screws and the skull surface was sealed by dental acrylic (Paladur, Heraeus Kulzer GmbH). One of the screws also served as the electrical ground. The wound was dusted with chlorhexidine antiseptic powder (Riemser, Germany). The animals were treated before and after surgery with analgesia (Meloxicam, Vetmedca GmbH, Ingelheim, Germany). The animals were allowed at least 7 days to recover from surgery before screenings were conducted. During this period, they were monitored closely for infection or distress and handled regularly.

### Single-unit recordings

Rats were screened once or twice daily for unit activity in a screening box that was visually distinct from and in a different room than the test arena. Neural activities were passed through AC-coupled, unity-gain operational amplifiers, which were mounted on a headstage (Axona, UK) connected close to the rat’s head through a socket that fitted onto the microdrive plug. The headstage was linked to a pre-amplifier via lightweight hearing-aid wires. The buffered signal from the headstage was amplified 6000–30,000 times in the pre-amplifier and then digitized (48 kHz) and bandpass filtered (0.6–7 kHz) in the dacqUSB system unit (Axona, UK). Each tetrode could be recorded differentially, being referenced by one electrode of another tetrode. One of the recording channels was dedicated to EEG recordings. The position of the rat was monitored by a video camera mounted directly above the arena and converted into *x*-*y* coordinates by a tracking system that detected a small light mounted on the headstage near the rat’s head.

### Behavioral apparatus

All screening for units took place in an open field square box (80 × 80 cm) with walls that were 70 cm high. When well-isolated place cells with stable fields were confirmed, experiments were performed in a circular arena (80 cm in diameter, 70 cm wall height) to circumvent that somatosensory cues derived e.g., from corner detection could assist in navigation/orientation. In addition, the walls and floor were made with smooth plastic surfaces so that no sensory cues/texture could be perceived from differences in texture. A cue card with a patterned image (60 cm width × 40 cm height) was placed on the wall, serving as an orientation landmark. The cue was placed high enough to avoid being a tactile landmark in darkness. A very dim red light (6 W) was only turned on while the rats were being connected to the recording system and turned off before they were randomly placed into the recording arena: Wistar rats are unpigmented and cannot see in red light. The recording setup and recording arena were both located in the same room. All light sources were turned off, or covered with opaque lightproof tape during recordings. An acoustic white noise generator was placed just underneath the arena to mask auditory cues that the rats could use for orientation. The floor and walls of the arena were cleaned between trials to remove and disperse olfactory cues. The experimental room had no windows. Recordings were thus performed in complete darkness in the absence of reliable external sensory cues.

### Experimental protocol

**Figure 1:**
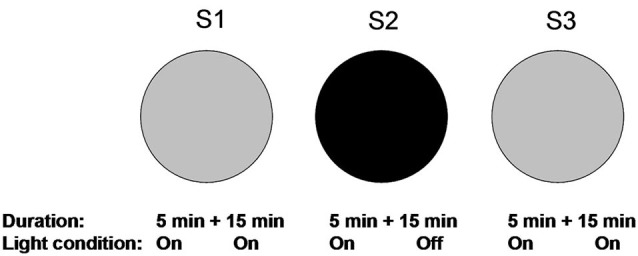
**Overview of the experimental paradigm**. Recording trials for the spatial drift paradigm are illustrated. Animals were habituated to the familiar circular arena one day before testing to make sure that stable place fields were established based on the visually perceived environment. On the experimental day, animals were first allowed to explore the circular arena under illuminated conditions. Recordings were initiated 5 min after entry and lasted for 15 min in the light (S1). In the second trial, animals re-entered in the arena in under illuminated conditions. The lights were turned off after 5 min and recordings were conducted for 15 min (S2). The condition for the last trial was the same as the first trial (S3). After each recording trial, animals were removed from the arena and the floor was cleaned.

The experimental protocol is shown in Figure 1. One day before the experiment started, animals were allowed to explore the circular arena twice, each for 15 min. Place fields tend to become stabilized within 15 min (Wilson and McNaughton, 1993). Place fields were recorded and confirmed as stable by these two trials. On the experimental day, animals were first allowed to explore the arena again for 5 min. This was to minimize unstable cell firing during the first several minutes by entering an environment (Wilson and McNaughton, 1993). After a brief confirmation of cell stability, recordings were continued for 15 min (S1). Thereafter, the animals were removed and returned to their home cages for 10 min, during which the floor and the walls were carefully cleaned to eliminate any local olfactory cues. This was to minimize the factor that local olfactory cues could be used for spatial navigation in darkness (Save et al., 2000; Zhang and Manahan-Vaughan, 2013). Next, animals were entered into the arena in illuminated conditions and were allowed to explore the arena for 5 min. During these 5 min, we checked whether urine or feces were left in the arena, which could be used as olfactory cues during subsequent navigation. If they occurred, animals were removed and the arena was carefully cleaned. If no olfactory cue was spotted, all lights were switched off while the animals remained in the arena. Recordings were then conducted for 15 min in darkness (S2). After S2, animals were again removed and returned to their home cages for 10 min. Lights were turned on again and the floor was cleaned. Finally, animals re-entered the arena with lights on. Recording was started after an initial exploration of 5 min and sustained for 15 min.

### Data analysis

Data analysis was performed using the Tint analysis software (Axona, UK). The collected waveforms were displayed as clusters by plotting the peak-to-peak amplitude of each spike on one electrode against that on each of the other three. The clusters were isolated by hand. On the basis of spike shape, firing rate, and firing location, complex spike cells were separated that had one or two firing fields. At least 50 spikes were isolated for each cluster. After the cluster cutting, firing rate maps for each cell were visualized using Tint, which divided the camera view arena into 64 × 64 square bins with a side length of 2.5 cm. The firing rate for a given cell in each bin indicated the spike number divided by dwell-time in that bin. The firing rate maps were smoothed and presented in color with the lowest firing rate (i.e., 0 Hz) in blue and the highest in red. A place field was defined as a contiguous group of pixels possessing a firing rate higher than half of the peak firing rate and covering less than 60% of the size of the recording arena. If a place cell was identified with one or more place fields, recordings were repeated 2–3 times on the same day and at least once more on the second day to verify its stability. If no qualified cell activity was identified, the tetrodes were advanced 25–50 µm and rats were returned to their home cages for at least 2 h. The maximum movement of tetrodes per day was 150 µm.

For each place cell, the smoothed firing rate map for each trial was examined to determine: (1) place field size; (2) average firing rate; (3) peak firing rate; (4) mean infield firing rate; (5) mean outfield firing rate; (6) spatial information content; (7) sparsity index; and (8) spatial coherence. The size of the place field was calculated as the percentage of the recording arena by the place field. The average firing rate was determined by dividing the number of spikes that occurred over the entire trial by the duration of the trial. The peak firing rate was the highest firing rate of all pixels within the place field of the cell. Mean infield and outfield firing rates were defined as the mean values for the firing rates of all pixels within (infield) and outside (outfield) the place field. The spatial information content, measured in bits/spike, is a measure of how much information about the spatial location of the animal is contained within the activity of the cell. It was calculated using the methods described by Skaggs et al. (1993). Sparsity is a measure of how compact the place field is relative to the recording arena and was calculated according to the methods described by Jung et al. (1994). The more confined the firing field of the place cell, the lower the sparsity index. There is a strong correlation between information content and sparsity. The similarity between two firing rate maps of neighboring trials was analyzed using a correlation procedure as follows. Each map was decomposed into a 32 × 32-element matrix. All pixels in one matrix were correlated, by a Pearson’s correlation, with their corresponding pixels in the second map. Pixels with a zero firing rate in both matrices were discarded. Spatial coherence is a measure of how spatially contiguous the neuron’s activity is. It was calculated in steps as follows. The firing rate map matrix was smoothed following a boxcar averaging procedure in which each pixel was replaced by the average over itself and its eight neighbors. The smoothed data set then was correlated to its unsmoothed form. This returned a Pearson’ *R*-value. The coherence was represented by a *Z*-score, which was calculated by Fisher’s transform following the equation: *Z* = 0.5 ln ((1+*R*)/(1-*R*)).

In order to exam the intra-trial stability of place fields, each trial (15 min) was divided into three segments (each for 5 min). Firing ratemaps generated in the first segment were cross-correlated to those generated in the second segment. Firing ratemaps generated in the second segment were cross-correlated to those generated in the third segment. Afterwards, correlation coefficients were compared between different recording conditions by analysis of variance (ANOVA).

### Trajectory slicing

To examine the impact of encountering the border of the environment on navigation, we analyzed the trajectory and spiking data gathered during the trials using a custom Python script. The script separates the individual segments of a given trajectory that lie in-between two consecutive border-contacts and exceed 10 cm in length. These segments are then cut in half to create a *from-border* and a *towards-border* slice of the given path segment. This trajectory slicing allows us to map and evaluate the spiking behavior of cells when the animal just encountered the border and compare it to when the animal is approaching a border and hence did not encounter one for a short while.

### Cell activity maps

The Python script was used to create different activity maps for each cell in all trial conditions– initial lights-on, lights-off, and lights-on-again phases– which yield nine maps per cell: (overall cell activity; activity of the cell over all from-border-slices; activity of the cell over all towards-border-slices) × (light-on; light-off; light-again conditions). An activity map was created by collecting all spikes of a given cell over the specified set of trajectory segments and sorting them into the bins of a regular 32 by 32 grid spanned over the whole environment. The accumulated values were then normalized according to the initial path’s slicing-criteria, which was chosen to be trajectory length in place or time. The resulting maps were plotted along the usual jet-scale to produce firing activity plots, and were compared to each other to determine significant differences. To compare two such maps, their 2D activities were first flattened into one dimensional vectors, which could then be compared using common metrics. We experimented with the Euclidian inner product, the Euclidian distance, the cosine of the angle between the vectors, and the standard correlation coefficient (Note that the first two of these measures are sensitive to the overall activity scaling, while the last two are not). For the results described here, we opted to use the cosine of the angle, since it seemed to produce the most stable metric over all the correlation experiments we assessed.

### Surrogate data

To be able to assess the significance of differences between two activity maps, we created additional surrogate data as a neutral reference for the observed cell data. It is based on the initial default conditions of the rats foraging with the lights still switched on. The algorithm to create the data iterates all wall-to-wall path segments and randomly swaps the actual from- and towards-border designation for the slices of each segment. This creates a randomized dataset with the statistics of the original data. The surrogate data is then used to create new activity maps based on the shuffled trajectory data. Those maps can then in turn be compared to each other, and we are able to determine a 5% significance threshold value which allows us to state that activity maps that differ a greater amount than this threshold value differ significantly from each other.

### Statistical analyses

A normality test (Kolmogorov-Smirnov) was applied to each data set before any other comparison to examine whether the data matched the pattern expected if the data were drawn from a population with a normal distribution. One-way analysis of variance (ANOVA) with repeated measures was applied to test significances through all trial-pairs. If the data were not normally distributed, Friedman ANOVA tests were conducted instead of one-way ANOVA, which were followed by *post hoc* tests. Statistical significance was set as *p* *≤* 0.05.

### Histological analysis

The location of the recording and stimulation electrodes was verified by postmortem histological visualization. The tissue was fixed, then coronal slices were obtained and Nissl stained (Manahan-Vaughan et al., 1998). Animals with misplaced electrodes were not included in the data analysis.

## Results

**Figure 2:**
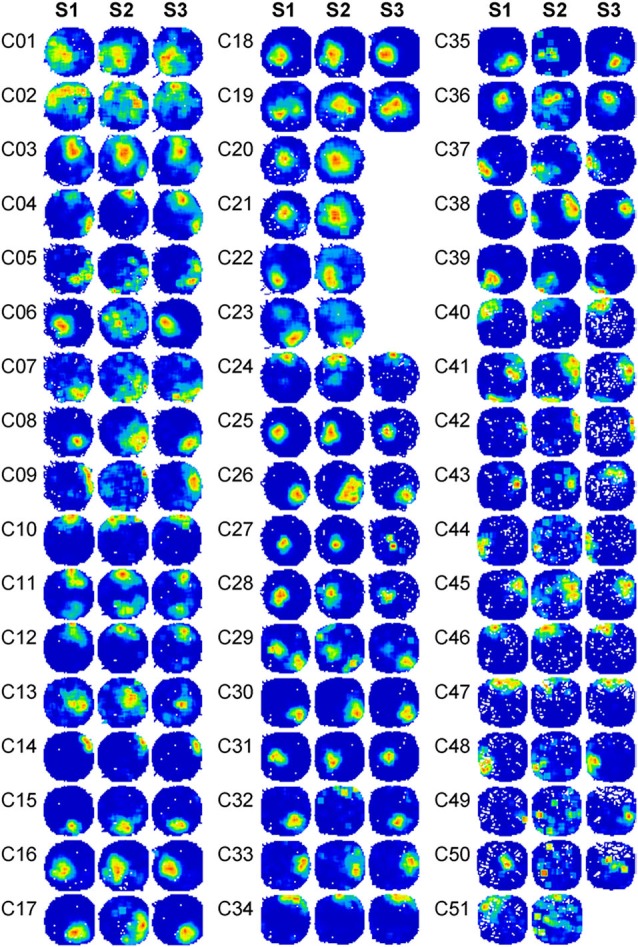
**Firing rate maps of 51 place cells in the spatial drift paradigm reveal changes in place cell firing**. Firing rate maps of 51 place cells across the spatial drift paradigm are illustrated. Recordings were conducted in a circular arena. Animals were allowed to explore the arena in the sequence of light-dark-light. Three firing rate maps were shown for each cell and were marked with different trial numbers. Trials S1 were recorded under illuminated conditions. Trials S2 were recorded in the dark. Trials S3 were recorded under illuminated conditions again. Before each recording trial started, animals always re-entered into the arena under illuminated conditions and were allowed to explore the arena for 5 min. Firing rate maps in S3 are not shown for five cells (C20, C21, C22, C23, and C51) due to insufficient movement of the animals during the recording period.

Fifty-one well-isolated place cells were recorded in the CA1 region of the hippocampus from four rats, following the sequence of light (S1), dark (S2), and light again (S3). They are shown in Figure 2. Place cells were recognized and selected using the criteria described in the Materials and Methods section. In Rat 1, 19 cells were detected in 5 recording sessions. In Rat 2, 17 cells were detected in 4 recording sessions. In Rat 3, 7 cells were detected in 3 recording sessions. In Rat 4, 8 cells were detected in 2 recording sessions. Five place cells (C20, C21, C22, C23, and C51) were not successfully recorded in the last trial (S3) as a result of poor behavior status. They were not included in statistical analyses involving the last trial. In each session, simultaneously recorded cells did not show similar behavior in register. For instance, the simultaneously recorded cells, C31 and C36, exhibited different responses in darkness, whereby cell C31 was almost invariant in the dark, whereas the stability of cell C36 became totally degraded.

**Figure 3:**
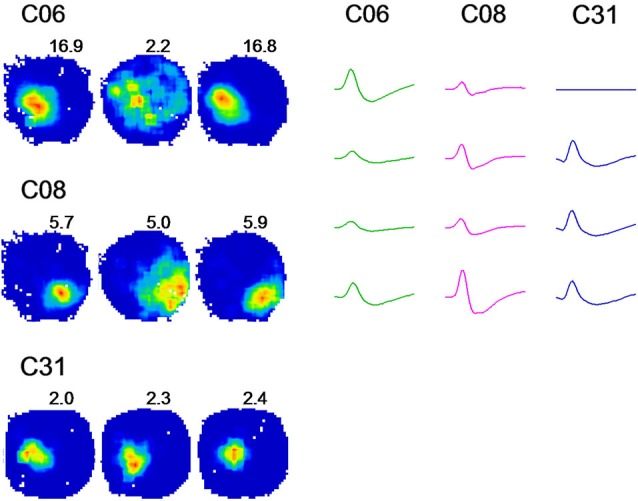
**A change from light to darkness reveals distinct firing rates in place cells**. Firing rate maps of 3 cells in the spatial drift paradigm are illustrated. Recordings were conducted in a circular arena. For each cell, three firing rate maps are shown with regard to three recording conditions: light-dark-light, respectively. Peak firing rates are indicated at the upper right region of each rate map. Analogs of spike waveforms of corresponding cells are illustrated for a time window of 2 ms. Cell C06 showed a strong reduction in firing when the lights were turned off, including reduced peak firing rates and increased firing fields. Cell C31 revealed minor changes upon the same environment change. The changes were shown only by a small number of cells, whereas the pattern of activity shown by C08 was expressed in the majority of place cells recorded. This cell showed small but notable increases in place field sizes and decreases in the peak firing rate.

We observed that each place cell responded to environmental changes in various ways. Therefore, three cells (C06, C08, and C31) were selected as representatives showing three typical changes of place fields in the dark (Figure 3). C06 exhibited dramatically degraded firing patterns when the lights were turned off, with a sharply reduced peak firing rate and increased firing field. C31 showed minor changes upon the same environmental change. Both extreme changes were noticed within a small number of cells, whereas the majority of cells (represented by C08) showed activity changes somewhere in between that of C06 and C31, with small but noticeable increases in place field sizes and decreases in peak firing rate occurring in the dark.

### Place field stability degrades in darkness

**Figure 4:**
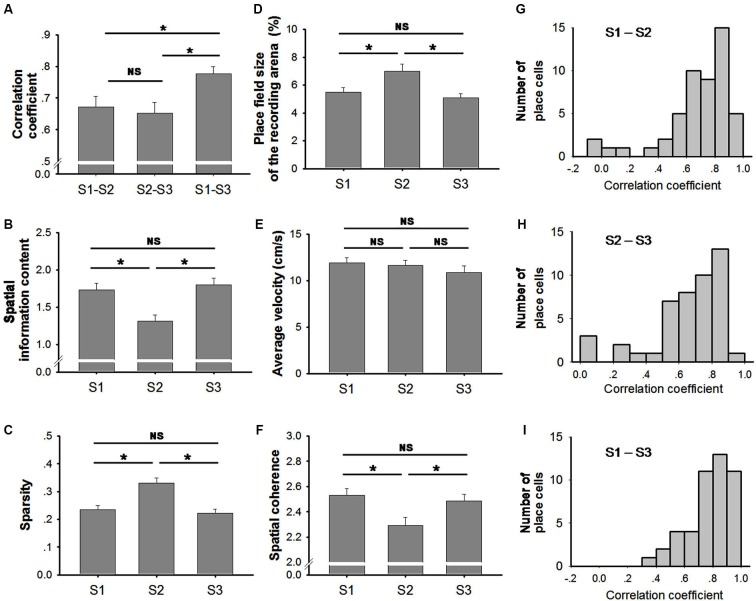
**Characteristics of place fields. (A)** Spatial correlations were significantly higher between S1 and S3 (two trials conducted in light conditions) than the other two trial-pairs (Friedman ANOVA + *post hoc* tests; * *p* < 0.05). No significant difference was observed between S1-S2 and S2-S3. This suggests that the stability of place fields decreased in the dark. **(B)** Spatial information was significantly lower in trial S2 (conducted in the dark) than in the other two trials (conducted in the light) (Friedman ANOVA + *post hoc* tests; * *p* < 0.05). No significant difference was observed between S1 and S3 (both trials conducted in the light). **(C)** Sparsity was significantly higher in trial S2 (conducted in the dark) than in the other two trials (conducted in the light) (Friedman ANOVA + *post hoc* tests; * *p* < 0.05). No significant difference was observed between S1 and S3. **(D)** Place field size was significantly higher in trial S2 (conducted in the dark) compared to the other two trials (conducted in the light) (Friedman ANOVA + *post hoc* tests; * *p* < 0.05). No significant difference was observed between S1 and S3 (both trials conducted in the light). **(E)** The average velocity of the animals in each trial did not vary significantly (One Way Repeated Measures ANOVA tests). **(F)** Spatial coherence was significantly lower in trial S2 (conducted in the dark) than that in the other two trials (conducted in the light). No significant difference was observed between S1 and S3 (Friedman ANOVA + *post hoc* tests; * *p* < 0.05). **(G–I)** Histograms are shown illustrating the distributions of correlation coefficients between trial S1 and S2 (G, light–dark) between trial S2 and S3 (H, dark– light); and between S1 and S3 (I, light–light).

The place cells exhibited stable firing patterns in both trials recorded in the light (S1 and S3). The correlation between the firing rate maps of the two trials indicated a high stability (mean ± SEM: 0.78 ± 0.02; Figure 4A). In contrast, spatial correlations of S1–S2 and S2–S3 both indicated a small but significant reduction (mean ± SEM: 0.68 ± 0.03 between S1–S2; 0.65 ± 0.03 between S2–S3; Figure 4A) (Friedman ANOVA + *post hoc* tests: Chi-square = 24.043). The reduction of spatial correlation suggests that the spatial stability was significantly degraded in the dark.

Given that individual place cells vary greatly in response to environmental changes, the distribution of spatial correlations are shown in histograms (Figures 4G–I). Spatial correlation coefficients were mostly clustered in high values ranging from 0.7 to 1, suggesting a high place field similarity between the two trials in light conditions (Figure 4I). In comparison, spatial correlation coefficients between light and dark conditions (Figure 4G) or between dark and light conditions (Figure 4H) were distributed more diffusively. Furthermore, both distributions of correlation coefficients in light-dark or dark-light conditions (Figures 4G,H) were extending to the left compared to that in light-light condition (Figure 4I).

In addition to the correlation coefficient, degraded place cell firing was also reflected in decreased spatial information content (Figure 4B) and an increased sparsity index (Figure 4C). The spatial information content was significantly lower in S2 (mean ± SEM: 1.31 ± 0.08) in comparison to that in S1 (mean ± SEM: 1.73 ± 0.09) and S3 (mean ± SEM: 1.80 ± 0.09) (Friedman ANOVA + *post hoc* tests: Chi-square = 40.217). This suggests that place cell firing is less accurate in the dark. The sparsity index was significantly higher in S2 (mean ± SEM: 0.33 ± 0.02) compared to that in S1 (mean ± SEM: 0.24 ± 0.01) and S3 (mean ± SEM: 0.22 ± 0.01), which indicated a more diffusive firing pattern of place cells in the dark (Friedman ANOVA + *post hoc* tests: Chi-square = 43.348). Taken together, our data suggest that navigation in the absence of visual input results in a small but significant degradation of hippocampal spatial firing pattern.

### Increased place field size and decreased peak firing rate occur as a result of drifted place cell firing

**Figure 5:**
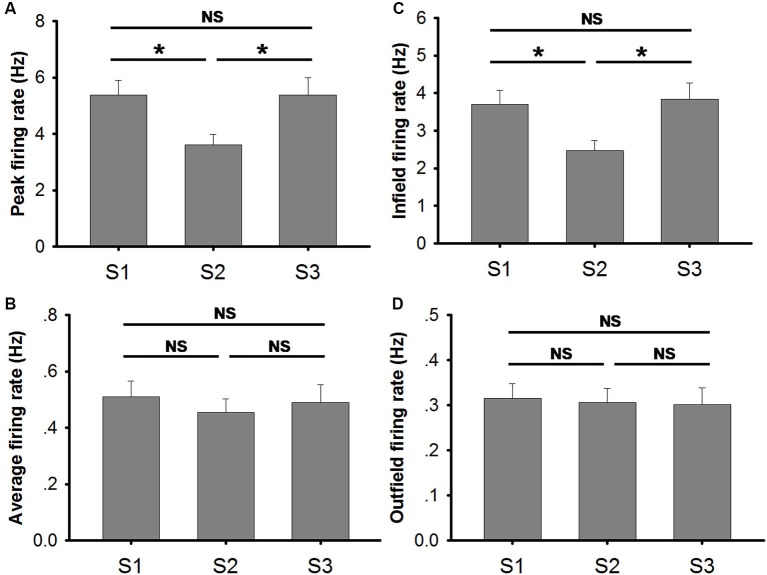
**Measures on firing frequency in conditions of light (S1), dark (S2) and light again (S3). (A)** Peak firing rate was significantly lower in trial S2 (conducted in the dark) than in the other two trials (conducted in the light). No significant difference was observed between S1 and S3 (Friedman ANOVA + *post hoc* tests; * *p* < 0.05). **(B)** The average firing rates of place cells in each trial are illustrated. No significant difference was observed (Friedman ANOVA test). **(C)** Infield firing rates of place cells in each trial are illustrated. Infield firing rate was significantly lower in trial S2 (conducted in the dark) than in the other two trials (conducted in the light). No significant difference was observed between S1 and S3 (Friedman ANOVA + *post hoc* tests; * *p* < 0.05). **(D)** Outfield firing rates of place cells in each trial are illustrated. No significant difference was observed (Friedman ANOVA test).

Place fields became larger when the lights were turned off (Figure 4D). Our data indicate a significant increase (~30%) in place field size in S2 (mean ± SEM: 7.00 ± 0.52) in comparison to that in S1 (mean ± SEM: 5.50 ± 0.33) and in S3 (mean ± SEM: 5.09 ± 0.30) (Friedman ANOVA + *post hoc* tests: Chi-square = 14.811). The increased place field size in S2 was coupled with a decrease in the peak firing rate (Figure 5A). The peak firing rate in S2 (mean ± SEM: 3.60 ± 0.39) was significantly lower than that in S1 (mean ± SEM: 5.37 ± 0.53) and S3 (mean ± SEM: 5.70 ± 0.63) (Friedman ANOVA + *post hoc* tests: Chi-square = 13.435). However, in contrast to peak firing rates, the average firing rate remained stable through all trials (Figure 5B). No significant difference was observed between trials (Friedman ANOVA: Chi-square = 1.696). This suggests that place cells do not fire more spikes when they lose visual input in the dark. The increased place field size and decreased peak firing rate leads to a possible assumption that the firing peak of a place cell in darkness drifts within the place field.

Moreover, our data also show that infield firing rate was significantly lower in S2 (mean ± SEM: 2.47 ± 0.27) than that in S1 (mean ± SEM: 3.70 ± 0.34) and S3 (mean ± SEM: 3.84 ± 0.44) (Friedman ANOVA + *post hoc* tests: Chi-square = 15.174) (Figure 5C). The decreased infield firing rate in S2 might be the outcome of increased place field size and unchanged average firing rate. The infield firing rate was calculated by dividing the spike number in the place field by the time animals stayed in the field. Spike number within the place field was, to a large extent, similar to the total spike number since most spikes occur within the field. The increased place field size implies a larger time base which is not compensated by larger spike number, hence, the low infield firing rate in S2 resulted. The outfield firing rate remained stable through all trials (Figure 5D). No significant difference was observed between trials (Friedman ANOVA: Chi-square = 0.696).

Place cell firing rate is positively correlated to the animals’ running speed (McNaughton et al., 1983; Wiener et al., 1989; Zhang et al., 1998). Our data suggest that the animal’s velocity was not affected by the light condition in the environment within the recording period (Figure 4E). Therefore, decreased peak and infield firing rate in the dark are not caused by reduced velocity but more likely correspond to drifting of place cell firing.

### Smoothness of place fields degrades in darkness

Spatial coherence is a measure that determines the “smoothness” of a place field. High spatial coherence values indicate consistent firing patterns of the place cells with stable firing peaks. Our data show that spatial coherence in S2 (mean ± SEM: 2.29 ± 0.07) was slightly but significantly decreased in comparison to that in S1 (mean ± SEM: 2.53 ± 0.05) and S3 (mean ± SEM: 2.49 ± 0.05) (Friedman ANOVA + *post hoc* tests: Chi-square = 15.826) (Figure 4F).

### Cross-correlation analysis indicates reduced intra-trial stability in darkness

**Figure 6:**
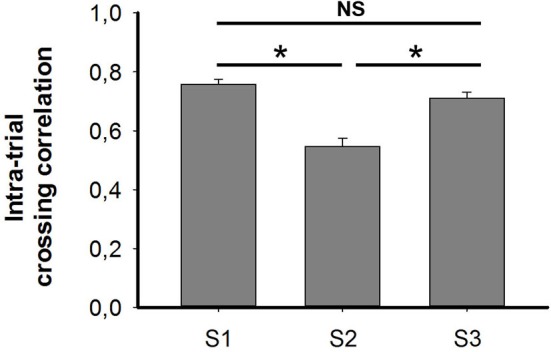
**Cross-correlations under conditions of light (S1), dark (S2) and re-exposure to light (S3)**. Cross-correlation coefficients were significantly lower in the dark (S2) than those conducted in the light, i.e., S1 and S3 (Friedman ANOVA + *post hoc* tests; * *p* < 0.05). No significance was observed between two trial conducted in the light. This suggests that the intra-trial stability of place fields was impaired in the dark due to drifts in place cell firing.

Each trial lasted 15 min and was divided into three temporally contiguous segments (5 min). The firing ratemap generated in each segment was cross-correlated to its previous, or to its subsequent, segment. Correlation coefficients in each trial were pooled together and are shown in the histogram in Figure 6. Cross-correlation coefficients in S2 (mean ± SEM: 0.54 ± 0.03) were significantly lower than those in S1 (mean ± SEM: 0.76 ± 0.02) and S3 (mean ± SEM: 0.71 ± 0.02) (Friedman ANOVA + *post hoc* tests: Chi-square = 32.435). No significant difference was found between S1 and S3, which comprised two trials that were conducted under illuminated conditions. The reduction of cross-correlation in S2 suggests an impaired intra-trial stability when animals were running in the darkness, that occurred due to drifting of place cell firing.

### Place cell firing in darkness is supported by border information

**Figure 7:**
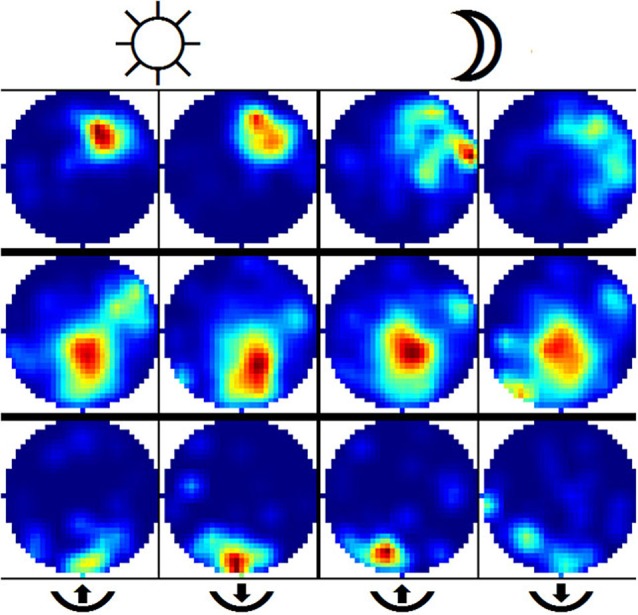
**Example of place cells illustrating firing patterns from from-border and towards-border segments**. Each row depicts the activity of one cell during under illuminated conditions (two leftmost columns) and in darkness (two rightmost columns). The firing activity is further split into *from-border* (columns no. 1 and 3) and *towards-border* segments. Activity in both *from* and *towards* cases was expected to stay largely the same with lights on and differ once lights are switched off. The second row depicts a cell where this was not the case and the rat was able to successfully detect its position during darkness. In contrast, the third row depicts a cell which features successful self-localization after just having encountered a wall but failing to do so when no wall was encountered for some time.

**Figure 8:**
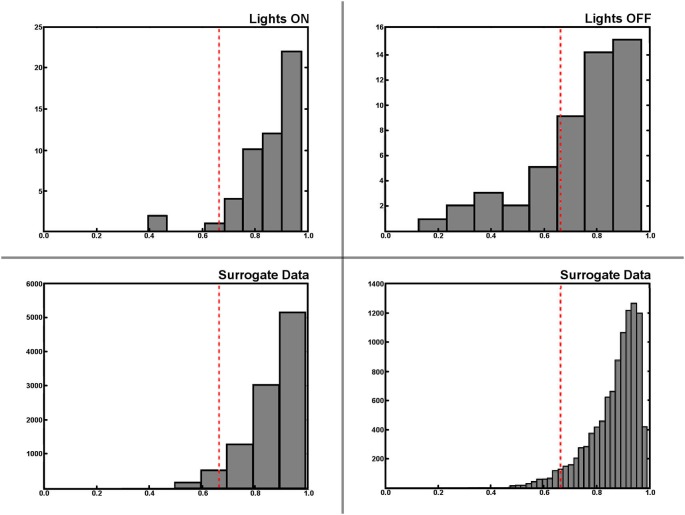
**Statistical analysis of place fields generated from from-border and towards-border trajectory segments**. Histograms of the differences between from-border and towards-border labeled trajectory segments during light and darkness (upper row). Note that differences between firing activity maps are measured as the cosine of the angle between two firing maps, and thus lower values indicate a larger difference between maps. The lower row shows the histogram of the same difference value being based on a large set of semi-randomly generated surrogate data instead of the original experimental recordings. The surrogate data was used to find the threshold value (dashed red line in all plots) determining an actual significant difference between two from-border/towards-border activity maps. The lower right plot shows this surrogate data in a higher resolution, while the lower left depicts the same data in the resolution as the histograms of the original data above– where it can be seen that 13 of 51 (25%) cells feature a significant different in their from-border/towards-border firing behavior in darkness, while the same effect seems to be restricted to mere outliers during the lights on condition.

Encountering the border of a known environment in darkness has a significant effect on the self-localization of rats. We split the trajectory into segments that were labeled as either *from* or *towards* (the border), depending on whether the rat was either traveling away from its last wall encounter or traveling towards its next wall encounter (see Section Materials and Methods for details on this, and the following procedures). This partitioning also allows the labeling of each neuronal spike as either *from* or *towards*, and as such the differences in firing behavior among those two states of travel can be observed. With the lights being switched on, not much difference is expected here as visual information can easily be used by the animals to locate themselves. In darkness, however, we would expect to find a difference between the two firing patterns in disoriented animals. Figure 7 shows a selection of *from/towards* activity from a small number of cells in light and darkness where this behavior is confirmed. To determine whether these differences in firing patterns are actually significant, we created an additional batch of surrogate data based on a large number or randomized permutations of our original recordings. This allows us to find a 5% threshold for differences between firing patterns featuring the natural statistics of the experimental data. Figure 8 shows this data in the form of histograms, where it can be seen that 13 (25%) out of the 51 recorded cells feature a significant difference in their firing pattern between from-border and towards-border states. Since contact with the wall in a completely circular environment cannot be used to correct orientation, but rather only serves to correct an error in the radial dimension, we attempted to further split up the measured error into an azimuthal and a radial component. Visual inspection of these data seemed to confirm a similar effect, but unfortunately its statistics were too weak to enable us to confirm it.

## Discussion

The idea that animals are able to keep track of their positions and find their way back home by means of integrating self-motion information was first postulated as far back as the 19th century (Darwin, 1873). In rodents, path integration was proposed as a mechanism to enable this (Mittelstaedt and Mittelstaedt, 1980) and this possibility was supported by observations that place fields are mostly preserved when visual landmarks in a familiar environment are suddenly removed (Muller and Kubie, 1987; O’Keefe and Speakman, 1987). Nonetheless, the orientations of place fields were sometimes found to have become randomly shifted by the time animals were returned to the (landmark-free) environment. Other studies have shown that place fields remained stable when the room lights were turned off (McNaughton et al., 1989; Quirk et al., 1990), if animals were first allowed to explore the apparatus under illuminated conditions. These findings suggest that spatial representations can, to a large extent, be maintained in the absence of visual input. This occurs possibly through a path integration mechanism.

However, other studies gave rise to more inconsistent results in terms of whether the path integration system remains stable and reliable in darkness. Head direction cell firing becomes compromised after about 2 min without visual inputs (Mizumori and Williams, 1993; Goodridge et al., 1998; Clark and Taube, 2011), suggesting that path integration suffers from drifting directional input immediately after losing external sensory input. However, grid cells, which receive inputs from head direction cells, exhibit stable firing patterns in the dark for up to about half an hour after onset of darkness (Hafting et al., 2005). Although one report indicates that place cells fire stably in the dark on the basis of idiothetic information (Quirk et al., 1990), another report suggests that idiothetic information is not sufficient for the maintenance of place fields in the absence of external cues (Save et al., 2000). All of these inconsistent observations lead to an assumption that path integration and uncontrolled external stimuli might mutually affect one another, and that the different observations with regard to path integration are due to the fact that external stimuli were controlled in a different manner in the different studies. A major challenge in behavioral tests of this kind is the effective (and undetected) removal of external cues, which could otherwise be used as spatial references. The way in which sensory cues were controlled during recordings might therefore have had a significant impact on the results. For example, both studies (Quirk et al., 1990; Save et al., 2000) used a similar paradigm in which animals were first exposed to an environment in illuminated conditions and then remained in the same environment in the dark. Local sensory cues derived from urine, stool or even hair deposits were not neutralized between the light and dark trials in the study by Quirk et al. (1990). This may have provided the animals with sufficient spatial information to ensure place field stability. In the study by Save et al. (2000), local traces were eliminated by cleaning the floor while animals were staying in the arena, before a recording period of 48 min in the dark. During cleaning, animals remained in the environment, however, meaning they may have been aware of the change in the environment. This form of cleaning may thus have affected place cell stability: on one hand, due to the loss of local olfactory traces or, on the other hand, as a result of the cleaning procedure and/or agent itself. In line with this possibility, remapping of place fields (or decreases in spatial correlations) occurs when the odor of the environment is changed (Anderson and Jeffery, 2003). Thus, the change in directed attention resulting from the cleaning of the environment in the presence of the rat could have altered place field firing. Accordingly, it has been reported that attention towards a spatially shifting olfactory cue compromises the stability of place fields (Muzzio et al., 2009). Therefore, current data on this topic do not permit a final conclusion whether path integration system alone is sufficient for maintenance of stable spatial representations.

In the present study, animals performed food exploration in a totally dark environment. White noise was applied to minimize possible auditory cues. The floor was cleaned between trials when the rat was absent from the arena. We observed that most of the place fields exhibited intermediate or minor degradation in darkness. Examples were seen in cells C06 and C08, for example, which were simultaneously recorded place cells. In our experimental design, animals were provided with a 5-min pre-exploration under illuminated conditions before recordings started. It was essential to allow animals to retrieve stable place fields under these conditions so that this data could be compared later to the place fields recorded in darkness. However, it can not be excluded that some local stimuli (e.g., hairs from the animals) were left in the arena during the 5 min exploration under illuminated conditions and that these were incorporated into the spatial representation and later used as spatial references in darkness. To circumvent this, recordings were terminated when urine or stools were left during the first 5 min so that these local stimuli were minimized.

Occasionally, the stability of the place fields began to degrade when the lights were turned off. An example of this can be seen in cell C36. Interestingly, the simultaneously recorded cells did not show a similar response in register. Hypothetically, place cells receive input from the head direction system and different place cells might be differentially wired to distinct head direction cells, which potentially could explain the effect, if the tuning of head direction cells drift incoherently. However, it has been reported that the preferred directions of head direction cells recorded simultaneously shift in register in darkness (Taube et al., 1990; Goodridge and Taube, 1995). Therefore, the varying degradation in darkness of simultaneously recorded place cells cannot be explained in this way.

The distribution of spatial correlations of firing rate maps between light and dark conditions was also examined. The data confirm our initial observation that most of the place fields exhibited intermediate or minor degradation in darkness. The majority of correlations between firing rate maps recorded under illuminated and dark conditions were higher than 0.5. The distribution of correlations became extended to the lower values in the dark. These observations all suggest a small, but significant, degradation of place fields in darkness. Place field size was also larger in the dark, but this does not mean that place cells become more active with a larger coverage of the active region. In fact, the average firing rate was unchanged whereas the peak firing rate was decreased. These data are in line with the hypothesis that place fields do in fact drift in darkness. Inter-trial spatial correlations indicated that degradation of place fields occurred in darkness. However, this effect could also result from a rapid remapping of place fields due to the loss of visual input when the light was turned off. Therefore, we also analyzed intra-trial place field stability. A significant reduction of intra-trial cross-correlation in the dark was found that strengthens our conclusion that place fields drift in darkness.

Although path integration is hypothesized to be based on self-motion without external feedback, most of the experiments on path integration could not avoid the fact that animals, running in an arena without visual, olfactory or auditory input, are still able to perceive the borders of an enclosure. Therefore, allocentric spatial representations could possibly be processed on the basis of border information. Actually, border information has been proven critical in place cell activity: place cells can cease firing, show multiple place fields or even break down, when the walls of a rectangular enclosure are removed sequentially in darkness (Barry et al., 2006). In particular, the most common response to wall removal was a dramatic breakdown of the place fields, especially for fields close to the removed wall rather than for fields more distant from the removed wall (Barry et al., 2006). In addition, a theoretical model has predicted that place field stability can be well maintained by a combination of path integration mechanism and border information in a circular environment without other external sensory input (Cheung et al., 2012). Moreover, place fields seem to be more stable in dark rectangular or radial arm environments than those in a dark circular environment (Markus et al., 1994). This suggests that irregularities in border information could help to strengthen spatial representations. In line with this, one would expect that path integration error is more likely to accumulate in circular environments.

In our study, we processed the trajectory within each recording trial into segments so that neuronal spikes of each place cell were categorized into two groups: spikes firing after a recent contact to the border and spikes firing before a border contact. We then compared the firing rate maps between these two states. A portion of cells showed different firing patterns before and after contact with the border. This suggests that border information contributes to place cell firing in darkness. Considering our first observation that different place fields diffuse in darkness to different degrees, even though they were simultaneously recorded, we examined whether cells responding to the border contact were simultaneously recorded, or whether all simultaneously recorded place cells respond to border contact in register. It was found that most of these cells were not simultaneously recorded and such kinds of responses were repeatable in different animals. It was also found that place cells do not react to border contact in register: during simultaneous recordings we observed cells that responded to the border contact and cells that did not. This suggests that place cells may behave individually with regard to their degradation in darkness, in contrast to head direction cells which drift in a consistent manner. This observation also suggests that place fields are unique in their degradation patterns in darkness, and whether they respond to boundary contact or not may relate to their particular role in encoding the position of the animal in the spatial environment.

As previously mentioned, irregularities in border information could strengthen spatial representations by giving more accurate positional cues. Therefore, a circular environment was chosen in this study so that contact with the border only offered positional updates in the radial dimension. We speculated at first that in darkness place fields would therefore only drift in the azimuthal dimension, but not in the radial dimension. Although we observed this phenomenon occasionally through visual observations, it was not confirmed by statistical tests (see Section Materials and Methods: Surrogate Data).

In conclusion, our data support the hypothesis that error accumulation in path integration drives place field drift in darkness. Thus, in the absence of reliable external sensory cues place fields became less stable and begin to drift during exploration of a circular arena in the dark. This drift is less severe if contact with the arena borders occurs.

## Conflict of interest statement

The authors declare that the research was conducted in the absence of any commercial or financial relationships that could be construed as a potential conflict of interest.

## References

[B1] Anderson M. I. Jeffery K. J. (2003). Heterogeneous modulation of place cell firing by changes in context. J. Neurosci. 23, 8827–8835 14523083 10.1523/JNEUROSCI.23-26-08827.2003 PMC6740394

[B2] Barry C. Lever C. Hayman R. Hartley T. Burton S. O’Keefe J. (2006). The boundary vector cell model of place cell firing and spatial memory. Rev. Neurosci. 17, 71–97 10.1515/revneuro.2006.17.1-2.71 16703944 PMC2677716

[B3] Cheung A. Ball D. Milford M. Wyeth G. Wiles J. (2012). Maintaining a cognitive map in darkness: the need to fuse boundary knowledge with path integration. PLoS Comput. Biol. 8:e1002651 10.1371/journal.pcbi.1002651 22916006 PMC3420935

[B4] Clark B. J. Taube J. S. (2011). Intact landmark control and angular path integration by head direction cells in the anterodorsal thalamus after lesions of the medial entorhinal cortex. Hippocampus 21, 767–782 10.1002/hipo.20874 21049489 PMC5723439

[B5] Darwin C. (1873). Origin of certain instincts. Nature 7, 417–418 10.1038/007417a0

[B5a] Etienne A. S. Jeffery K. J. (2004). Path integration in mammals. Hippocampus 14, 180–192 10.1002/hipo.10173 15098724

[B6] Goodridge J. P. Dudchenko P. A. Worboys K. A. Golob E. J. Taube J. S. (1998). Cue control and head direction cells. Behav. Neurosci. 112, 749–761 10.1037//0735-7044.112.4.749 9733184

[B31] Goodridge J. P. Taube J. S. (1995). Preferential use of the landmark navigational system by head direction cells in rats. Behav. Neurosci. 109, 49–61 10.1037//0735-7044.109.1.49 7734080

[B8] Hafting T. Fyhn M. Molden S. Moser M. B. Moser E. I. (2005). Microstructure of a spatial map in the entorhinal cortex. Nature 436, 801–806 10.1038/nature03721 15965463

[B9] Jung M. W. Wiener S. I. McNaughton B. L. (1994). Comparison of spatial firing characteristics of units in dorsal and ventral hippocampus of the rat. J. Neurosci. 14, 7347–7356 7996180 10.1523/JNEUROSCI.14-12-07347.1994 PMC6576902

[B10] Lever C. Burton S. Jeewajee A. O’Keefe J. Burgess N. (2009). Boundary vector cells in the subiculum of the hippocampal formation. J. Neurosci. 29, 9771–9777 10.1523/JNEUROSCI.1319-09.2009 19657030 PMC2736390

[B11] Manahan-Vaughan D. Behnisch G. Vieweg S. Reymann K. G. Behnisch T. (1998). Semi-automated analysis of NMDA-mediated toxicity in digitised colour images from rat hippocampus. J. Neurosci. Methods 82, 85–95 10.1016/s0165-0270(98)00042-9 10223518

[B12] Markus E. J. Barnes C. A. McNaughton B. L. Gladden V. L. Skaggs W. E. (1994). Spatial information content and reliability of hippocampal CA1 neurons: effects of visual input. Hippocampus 4, 410–421 10.1002/hipo.450040404 7874233

[B32] McNaughton B. L. Barnes C. A. Gerrard J. L. Gothard K. Jung M. W. Knierim J. J. (1996). Deciphering the hippocampal polyglot: the hippocampus as a path integration system. J. Exp. Biol. 199, 173–185 8576689 10.1242/jeb.199.1.173

[B13] McNaughton B. L. Barnes C. A. O’Keefe J. (1983). The contributions of position, direction and velocity to single unit activity in the hippocampus of freely-moving rats. Exp. Brain Res. 52, 41–49 10.1007/bf00237147 6628596

[B33] McNaughton B. L. Battaglia F. P. Jensen O. Moser E. I. Moser M. B. (2006). Path integration and the neural basis of the ‘cognitive map’. Nat. Rev. Neurosci. 7, 663–678 10.1038/nrn1932 16858394

[B14] McNaughton B. L. Leonard B. Chen L. (1989). Cortical-hippocampal interactions and cognitive mapping: a hypothesis based on reintegration of the parietal and inferotemporal pathways for visual processing. Psychobiol. 17, 230–235 10.1007/bf03337774

[B15] Mittelstaedt M. L. Mittelstaedt H. (1980). Homing by path integration in a mammal. Naturwissenschaften 67, 566–567 10.1007/bf00450672

[B16] Mizumori S. J. Williams J. D. (1993). Directionally selective mnemonic properties of neurons in the lateral dorsal nucleus of the thalamus of rats. J. Neurosci. 13, 4015–4028 8366357 10.1523/JNEUROSCI.13-09-04015.1993 PMC6576470

[B17] Muller R. U. Kubie J. L. (1987). The effects of changes in the environment on the spatial firing of hippocampal complex-spike cells. J. Neurosci. 7, 1951–1968 3612226 10.1523/JNEUROSCI.07-07-01951.1987 PMC6568940

[B18] Muzzio I. A. Levita L. Kulkarni J. Monaco J. Kentros C. Stead M. (2009). Attention enhances the retrieval and stability of visuospatial and olfactory representations in the dorsal hippocampus. PLoS Biol. 7:e1000140 10.1371/journal.pbio.1000140 19564903 PMC2696347

[B19] O’Keefe J. Dostrovsky J. (1971). The hippocampus as a spatial map. Preliminary evidence from unit activity in the freely-moving rat. Brain Res. 34, 171–175 10.1016/0006-8993(71)90358-1 5124915

[B20] O’Keefe J. Speakman A. (1987). Single unit activity in the rat hippocampus during a spatial memory task. Exp. Brain Res. 68, 1–27 10.1007/bf00255230 3691688

[B21] Quirk G. J. Muller R. U. Kubie J. L. (1990). The firing of hippocampal place cells in the dark depends on the rat’s recent experience. J. Neurosci. 10, 2008–2017 2355262 10.1523/JNEUROSCI.10-06-02008.1990 PMC6570323

[B22] Rochefort C. Arabo A. Andre M. Poucet B. Save E. Rondi-Reig L. (2011). Cerebellum shapes hippocampal spatial code. Science 334, 385–389 10.1126/science.1207403 22021859

[B23] Save E. Nerad L. Poucet B. (2000). Contribution of multiple sensory information to place field stability in hippocampal place cells. Hippocampus 10, 64–76 10.1002/(sici)1098-1063(2000)10:1<64::aid-hipo7>3.3.co;2-p 10706218

[B24] Skaggs M. E. McNaughton B. L. Gothard K. M. Markus E. J. (1993). An information-theoretic approach to deciphering the hippocampal code. Adv. Neural Inform. Process Syst. 5, 1030–1037

[B25] Solstad T. Boccara C. N. Kropff E. Moser M. B. Moser E. I. (2008). Representation of geometric borders in the entorhinal cortex. Science 322, 1865–1868 10.1126/science.1166466 19095945

[B26] Taube J. S. Muller R. U. Ranck J. B. Jr. (1990). Head-direction cells recorded from the postsubiculum in freely moving rats. II. Effects of environmental manipulations. J. Neurosci. 10, 436–447 2303852 10.1523/JNEUROSCI.10-02-00436.1990 PMC6570161

[B27] Wiener S. I. Paul C. A. Eichenbaum H. (1989). Spatial and behavioral correlates of hippocampal neuronal activity. J. Neurosci. 9, 2737–2763 2769364 10.1523/JNEUROSCI.09-08-02737.1989 PMC6569688

[B28] Wilson M. A. McNaughton B. L. (1993). Dynamics of the hippocampal ensemble code for space. Science 261, 1055–1058 10.1126/science.8351520 8351520

[B29] Zhang K. Ginzburg I. McNaughton B. L. Sejnowski T. J. (1998). Interpreting neuronal population activity by reconstruction: unified framework with application to hippocampal place cells. J. Neurophysiol. 79, 1017–1044 9463459 10.1152/jn.1998.79.2.1017

[B30] Zhang S. Manahan-Vaughan D. (2013). Spatial olfactory learning contributes to place field formation in the hippocampus. Cereb. Cortex [Epub ahead of print]. 10.1093/cercor/bht239 24008582 PMC4380081

